# Plasma Metabolites, Productive Performance and Rumen Volatile Fatty Acid Profiles of Northern Australian *Bos indicus* Steers Supplemented with *Desmanthus* and Lucerne

**DOI:** 10.3390/metabo11060356

**Published:** 2021-06-02

**Authors:** Bénédicte Suybeng, Edward Charmley, Christopher P. Gardiner, Bunmi S. Malau-Aduli, Aduli E. O. Malau-Aduli

**Affiliations:** 1 Animal Genetics and Nutrition, Veterinary Sciences Discipline, College of Public Health, Medical and Veterinary Sciences, Division of Tropical Health and Medicine, James Cook University, Townsville, QLD 4811, Australia; benedicte.suybeng@my.jcu.edu.au (B.S.); christopher.gardiner@jcu.edu.au (C.P.G.); 2 CSIRO Agriculture and Food, Private Mail Bag Aitkenvale, Australian Tropical Sciences and Innovation Precinct, James Cook University, Townsville, QLD 4811, Australia; ed.charmley@csiro.au; 3 College of Medicine and Dentistry, Division of Tropical Health and Medicine, James Cook University, Townsville, QLD 4811, Australia; bunmi.malauaduli@jcu.edu.au

**Keywords:** *Desmanthus virgatus*, *Desmanthus leptophyllus*, *Desmanthus bicornutus*, volatile fatty acids, plasma metabolites, legumes, tropical beef cattle, polyethylene glycol, nitrogen metabolism

## Abstract

The hypothesis tested was that tropical steers supplemented with the *Desmanthus* legume and lucerne, a widely characterized temperate legume of high nutritive value, would elicit similar responses in plasma metabolite profiles, productive performance, nitrogen retention, and volatile fatty acids (VFA). The tannin-binding compound, polyethylene glycol-4000 (PEG), was added to the diets (160 g/kg *Desmanthus* dry matter) with the objective of further exploring nitrogen (N) utilization in the animals supplemented with *Desmanthus* relative to lucerne. From February to June 2020, sixteen yearling Brangus steers (average liveweight of 232 ± 6 kg) were fed a background diet of Rhodes grass (*Chloris gayana*) hay for 28 days, before introducing three *Desmanthus* cultivars (*Desmanthus virgatus* cv. JCU2, *D. bicornutus* cv. JCU4, *D. leptophyllus* cv. JCU7) and lucerne (*Medicago sativa*) at 30% dry matter intake (DMI). Relative to the backgrounding period, all supplemented steers exhibited similar growth performance. Steers supplemented with *Desmanthus* recorded a lower DMI and animal growth performance, but higher fecal N concentration than animals supplemented with lucerne. Among the three *Desmanthus* cultivars, there were no significant differences in N concentrations, VFA, and plasma metabolite profiles. The addition of PEG induced higher rumen iso-acid concentrations and fecal N excretion. However, feeding *Desmanthus* spp. to tropical *Bos indicus* steers could be a valuable means of increasing N utilization, which is attributable to the presence of tannins, and, consequently, improve animal productive performance. Since supplementation with lucerne resulted in higher liveweight, daily liveweight gains, and overall animal performance than supplementing with *Desmanthus*, the tested hypothesis that both supplements will elicit similar animal performance does not hold and must be rejected. Further in vivo investigation is needed to better understand the impact of tannins in *Desmanthus* on N utilization.

## 1. Introduction

“Metabolomics” as a research discipline, is a term derived from “the study of metabolites”, which comprehensively measures the end-products (small molecule metabolites) of complex in vivo metabolic processes such as glucose, urea, non-esterified fatty acids (NEFA), bilirubin, aspartate aminotransferase (AST), etc., in cells, biofluids and tissues using advanced analytical chemistry techniques. The blood metabolome provides a suite of predictive biomarkers for livestock health, productive performance, and disease monitoring because cattle go through physiological and metabolic adjustments during growth and development [1,2]. Recent research investigations in metabolomics have generated compelling results showing that metabolites such as glucose and urea can help farmers and the livestock industry to evaluate dietary responses to different feeds, and this makes metabolomics an ideal tool for livestock research [2]. For instance, in fattening Holstein bulls, Yang et al. [3] reported that plasma ammonia (NH_3_) was a metabolic waste product and any increase in its circulation due to inefficient nitrogen (N) conversion to amino acids would affect animal health and growth performance. In Wagyu crossbred steers, Connolly et al. [4] found that blood metabolites were either positively or negatively correlated with key production traits including growth rate, carcass weight, and subcutaneous and intramuscular fat, thus potentially offering biomarkers that could be used for individual steer selection for feedlot performance. Whereas published reports on the impacts of seasonal and dietary nutrient supplementation on rumen microbiota structure and metabolites of beef cattle abound [5,6,7], to our current knowledge of the published literature, there are no existing peer reviewed reports on the plasma metabolite profiles of tropical northern Australian beef cattle steers supplemented with the tropical legume *Desmanthus.* Our present research was intended to fill this knowledge gap. 

The Northern Australian beef industry is defined by an extensive grazing system in dry tropical rangelands. In this particularly low N environment, undernutrition is the major issue, especially during the dry winter season [5]. Animals that efficiently convert feed into meat or body mass have a high difference between their actual feed intake and the expected feed requirements for maintenance and growth (low residual feed intake, RFI) over a particular time period [8]. A recent study demonstrated that feeding cattle with an N-supplemented diet in this environment enhanced rumen fermentation and increased bacterial populations involved in pectin and hemicellulose degradation and ammonia assimilation [5]. This change in the rumen microbiota structure induced a lower RFI with an increase in the daily LW gain, rumen NH_3_-N, butyrate, and the acetic to propionic acid ratio [5]. High quality feeds have been shown to increase plasma urea which serves as a promising and inexpensive metabolite to predict and categorize bovine RFI values [8,9]. Additionally, dietary proteins in roughages and N utilization are fundamental to growth and development in ruminants, but an estimated 70% of ingested N is excreted as urinary and fecal N which can limit animal productive performance and cause environmental pollution [3]. A decrease in fecal and urinary N implies a higher N retention and a more efficient regulation of the urea cycle and conversion of rumen NH_3_-N. Therefore, a better understanding of energy and protein metabolism through an assessment and synthesis of rumen volatile fatty acids (VFA), branched-chain fatty acids (iso-acids) and plasma metabolites in steers supplemented with *Desmanthus* relative to lucerne (*Medicago sativa*), will provide baseline information on NH_3_-N synthesis, urinary and fecal N excretion, and hence N retention and utilization for growth and liveweight (LW) gain. 

Lucerne is considered as one of the main perennial legumes in the world with an estimated world cropping area of 30 million ha (mainly located in North America, Europe, and South America), and an extensive use for ruminant livestock feeding systems in temperate Australia [10]. It is globally used due to its high quality crude protein (CP) content ranging between 14 and 24% on a dry matter (DM) basis [11,12,13,14], which induces an increase in animal production. McDonald et al. [11] stated that in the Australian southern state of New South Wales, sheep wool and cattle LW increased by 10–20% when lucerne was included in pastures based on subterranean clover or phalaris. Lucerne is not adapted to most central Queensland soils [15] because of its intolerance to saline soils [10] and failure to persist in sufficient plant densities for more than 2 years [15]. In contrast, a comprehensive review by Suybeng et al. [16] on the use of *Desmanthus* (JCU1 cv. *D. leptophyllus* and JCU4 cv. *D. bicornutus*) for beef cattle production in Northern Australia showed that *Desmanthus* as a tropical legume, not only persists and survives under harsh tropical conditions, but also contains high CP levels that elicited promising LW gains in steers, sheep, and goats. Kanani et al. [12] reported that supplementing goats with 40% *Desmanthus bicornutus* and 60% Sudangrass (*Sorghum bicolor*) induced a daily LW gain of 60.9 g compared to 82.3 g on 40% lucerne and 60% Sudangrass. However, *Desmanthus* contains tannins which are polyphenolic molecules that have the ability to form complexes with proteins, and to a lesser extent, with metal ions, amino acids, and polysaccharides [17]. Tannins have been shown to improve N utilization by decreasing rumen degradability of CP and sometimes CP digestibility in the digestive tract which shifts N loss from urine to feces [18,19,20]. Fecal N is mainly in the organic NH_2_ form which has to be mineralized to ammonium (NH_4_^+^) before it can volatilize or leach. The CT-protein complex inhibits this mineralization process by slowing down the breakdown of protein from feces to NH_4_^+^ [19]. More than 70% of urinary N is in urea form which is readily available for hydrolysis and conversion into NH_4_^+^ [21]. The nitrification of NH_4_^+^ to nitrate (NO_3_^−^) produces the gaseous forms of nitric oxide (NO), nitrous oxide (N_2_O), or dinitrogen (N_2_) with the reduction of N. Nitrous oxide may also be produced by denitrification where nitrite (NO_2_^−^) may be reduced to NO, N_2_O, or N_2_ instead of being oxidized to NO_3_^−^ [21]. Nitrogen excretion contributes to environmental pollution via NH_3_ or N_2_O volatilization from the soil surface or NO_3_ in the soil that may be leached into ground water [19]. Tannins were also described in the past as anti-nutritional factors due to their negative impact on animal nutrition such as lower feed intake, protein, dry matter and N digestibility, LW gains, milk yield, and wool growth [22]. However, polyethylene glycol (PEG) can bind to tannins and break the already formed tannin–protein complexes as their affinity for tannins is higher than for proteins [17]. A previous study on low quality feeds with 9.6% CP content showed that the addition of PEG in a diet containing 22% *Desmanthus* (JCU1 and JCU4) on a DM basis harvested at the end of the dry season did not have an impact on total VFA, daily LW gain, or dry matter intake (DMI), but increased rumen NH_3_-N and iso-acid concentrations [16]. As the anti-nutritional effects of phenolic compounds and tannins vary between species and seasons [23], it was considered important to evaluate *Desmanthus* cultivars at a higher dietary inclusion rate and harvested in the late wet season. The addition of PEG (160 g/kg *Desmanthus* DM) was to determine if tannins were affecting N utilization.

Therefore, the primary objective of this investigation was to evaluate the impact of supplementing Brangus steers on a basal diet of Rhodes grass with either the tropical legume *Desmanthus* spp. or the temperate legume lucerne on animal performance (DMI, LW gain), rumen VFA and plasma metabolite profiles, N intake, rumen NH_3_-N, blood urea, fecal N, and urinary N concentrations. This research tested the hypothesis that tropical steers supplemented with *Desmanthus* and lucerne legumes would elicit similar responses in plasma metabolite profiles, productive performance, and volatile fatty acids. 

## 2. Results

### 2.1. Chemical Composition

The nutrient compositions of Rhodes grass hay, lucerne hay, and the three *Desmanthus* cultivars are given in Table 1. Rhodes grass had a lower CP concentration and a higher fiber content than the three *Desmanthus* cultivars and lucerne. However, the Rhodes grass DMD was similar to the 3 *Desmanthus* cultivars which had a similar ME between Rhodes grass and the *Desmanthus* spp. JCU2 and JCU7 had similar compositions. Lucerne had a higher CP and lower fiber concentration than the three *Desmanthus* cultivars which resulted in a higher DMD and ME. The NIRS and wet chemistry CP, NDF, DMD, and ME values were highly correlated (Table 2). However, the NIRS and wet chemistry values were not significantly different in CP, ADF, DMD, and ME concentrations.

The nutritive values of lucerne and *Desmanthus* treatments are given in Table 3. CP contents were similar between treatments. The higher ADF content in the *Desmanthus* cultivars compared to lucerne induced a significantly lower ME in the *Desmanthus* diets.

### 2.2. Animal Performance

As the animals changed *Desmanthus* cultivars in each period to minimize variability, the animal performance was inferred from the average of the 3 *Desmanthus* spp. As shown in Table 4, at the start of the experiment, the DMI and LW of all animals were similar. At the end of the feeding trial, the animals fed lucerne had significantly higher DMI and LW than the animals fed *Desmanthus* spp. Therefore, the animals on the lucerne diet had a significantly higher daily LW gain than the animals on *Desmanthus* spp. diet. When the DMI was expressed as DMI/kg LW percentage, it was the same for animals fed lucerne and *Desmanthus* spp. Although the feed conversion ratio (FCR) of the animals on the *Desmanthus* spp. diet was higher than the FCR of the animals on the lucerne diet, it was not significantly different.

### 2.3. Effect of Lucerne and Desmanthus on Rumen and Plasma Metabolites

The concentration of iso-valerate and NEFA were significantly higher in animals fed lucerne compared to the animals on the JCU4 diet (Table 5). Total VFA concentration was significantly higher for the animals on lucerne diet compared to the ones on JCU7 and the concentration of iso-butyrate was significantly higher for the animals on lucerne compared to the ones on JCU4 and JCU7. There was no difference in acetate, propionate, acetate:propionate ratio, n-butyrate, n-valerate, n-caproate, glucose, or cortisol between the animals fed lucerne or *Desmanthus* spp. The pH was similar regardless of the type of legume supplement.

### 2.4. Nitrogen Metabolism

As depicted in Table 6, dietary N values from wet chemistry analyses were similar in *Desmanthus* spp. and lucerne, while predicted dietary N from F.NIRS analysis was higher in lucerne than in *Desmanthus*, resulting in a significantly higher N intake in animals supplemented with lucerne compared to JCU2 and JCU7 diets. Fecal N concentration was significantly lower in animals fed lucerne compared to those on *Desmanthus*. Between the cultivars, fecal N was significantly higher in animals fed JCU7 compared to those on JCU2 and JCU4. There were no differences in rumen NH_3_-N, plasma urea, or urinary N between the different diets. 

### 2.5. Effect of Polyethylene Glycol on Animal Performance, Rumen VFA, Plasma Metabolites, and Nitrogen Retention

The PEG effect was compared only between the animals fed *Desmanthus* (Table 7). The PEG addition had no effect on DMI, plasma metabolites, and N concentrations. Only the concentration of rumen iso-butyrate, iso-valerate, and n-valerate significantly increased with the PEG addition.

## 3. Discussion

The objective of this study was to compare three species of *Desmanthus*, a tropically adapted legume, with the well characterized and widely grown temperate legume, lucerne. Across most indices measured in this paper, the results for lucerne are consistent with the literature [11,12,13,14], confirming it to be a legume of high nutritive value. *Desmanthus* spp. were of a lower quality with higher fiber and lower energy content than lucerne which resulted in lower intake and performance. However, it should be noted that lucerne was fed as hay and was of consistent nutritive value throughout the trial. Securing and feeding a consistent and acceptable supply of *Desmanthus* over three months was challenging and this may have influenced the results. The anticipated temporal variation in chemical composition and nutritive value of *Desmanthus* necessitated the adoption of a randomized block design for the *Desmanthus* treatments, even if this incurred nutritional perturbations as animals shifted from one cultivar to another. 

### 3.1. Chemical Composition

NIRS predictions for 20 species of perennial legumes showed a good correlation with the wet chemistry results with an R^2^ > 0.7 for NDF, ADF, DMD, OM, ME, and N concentrations [26]. The lower correlation in the present study for the determination of ADF suggests that more calibration studies are needed to better predict these values for *Desmanthus*. The high correlation between the NIRS and wet chemistry data for NDF content suggests a strong relationship, but this observation should be interpreted with caution given the smaller sample size in this study compared to the 4385 samples analyzed by Norman et al. [26]. However, regardless of the method used to analyze the feed composition of the forage, lucerne was of a higher quality than the 3 *Desmanthus* cultivars with a higher CP and lower fiber content. 

### 3.2. Animal Performance

A previous study demonstrated a daily LW gain of 0.18 kg with 31% *Desmanthus* (JCU1 or JCU4) inclusion in the diet [16]. In our present study, a daily LW gain of 0.34 kg was obtained by supplementing steers with 30% *Desmanthus* on a DM basis. The higher daily LW gain can be explained by the higher quality of the *Desmanthus* and Rhodes grass resulting in higher DMI/kg LW (2% for the current study compared to 1.6% for the previous study). The significantly higher daily LW gain observed in the animals on lucerne compared to *Desmanthus* can be due to the higher feed quality of the lucerne treatment and a higher voluntary consumption of lucerne compared to the *Desmanthus* spp. Kanani et al. [12] compared intake and growth performance of goats fed Sudangrass supplemented ad libitum with either lucerne or *Desmanthus*, where the nutritive value was similar between the two legumes. Voluntary intake of *Desmanthus* was lower than for lucerne and this was reflected in lower LW gain. Sonawane et al. [27] replaced a concentrate diet with either 50 or 100% *D. virgatus* and showed that goats fed the 50% *Desmanthus* diet had the highest LW gain despite having a lower intake compared with the 100 or 0% *Desmanthus* diets. Our results broadly corroborate those of Kanani et al. [12] and Sonawane et al. [27] which suggest that performance of animals fed *Desmanthus*-containing diets was lower than in animals fed diets containing lucerne or concentrates. 

### 3.3. Effect of Lucerne and Desmanthus on Rumen Volatile Fatty Acids and Plasma Metabolites

Volatile fatty acids constitute the main source of metabolizable energy from rumen fermentation in ruminants [28]. In our study, the higher concentration of total VFA in the animals on lucerne compared to the animals fed JCU7 may be due to the significantly greater supply of protein-N, which once proteolyzed form amino acids and are deaminated, and VFA produced by fermentation of the carbon skeletons formed [29]. The higher ME in the lucerne diet coupled with additional VFA from amino acid catabolism was associated with an additional 260 g/d LW gain compared to cattle fed the *Desmanthus* diets. The difference in VFA concentration may also be associated with the presence of tannins in the *Desmanthus* spp. [30,31], although Suybeng et al. [16] showed previously that rumen VFA concentration was linearly correlated with an increasing level of *Desmanthus* in the diet. In the present study, the concentration of iso-butyrate was significantly higher in the rumen of animals fed lucerne than those on JCU4 and JCU7, while the concentration of iso-valerate was significantly higher for the animals fed lucerne than those on JCU4. The branched-chain VFA derived from branched-chain amino acids tend to increase with an increase in dietary N in the rumen [32]. Thus, the concentration of iso-acids was higher in animals fed lucerne than in the ones fed JCU4 and JCU7 due to the higher CP intake. This observation corroborates previous findings by Martinez–Fernandez et al. [5] who reported an increase in rumen iso-acids in cattle supplemented with N compared to the animals fed an un-supplemented diet. The lack of treatment effect on the main VFA proportions (acetate and propionate) suggests that *Desmanthus* spp. and tannins had no negative impact on rumen digestibility. However, total VFA (mg/100 dL) were affected by treatment, suggesting an impaired rumen digestibility of Lucerne vs. JCU7 diet. Similar to our observation, Aboagye et al. [33] and Aguerre et al. [34] did not find any effect of tannins on propionate and acetate in cattle. This observation was in contrast with a previous report by Beauchemin et al. [35] who found a linear decrease in acetate with an increase in quebracho tannin extract in growing beef cattle fed a forage-based diet with 16.0% CP. Suybeng et al. [16] reported a linear increase in acetate to propionate ratio with an increase in *Desmanthus* level in a 9.6% CP diet. This difference may be due to variation in diet quality and processing. The rumen pH in our study was within the range of the normal pH of the rumen fluid in cattle fed pasture diets (6.0–7.2) [36]. The absence of the effect of tannin treatments on pH corroborates the findings of other studies with tannins in cattle feeding trials [16,33]. 

Glucose and NEFA were within the range of normal metabolite concentrations found in cattle [37,38,39,40,41,42]. Russel and Wright [43] stated that plasma glucose concentration was not likely to constitute a useful index of energy intake or status (R^2^ = 0.04) in housed or grazing animals due to the insensitivity of circulating concentrations to nutritional change and its concurrent sensitivity to stress [44]. Clemmons et al. [45] also showed no difference in glucose concentrations between steers of low and high RFI. They attributed this lack of difference to the tight regulation of glucose in ruminants. On the other hand, plasma NEFA has been shown to be highly correlated with energy intake in the diet (R^2^ = 0.89) and is consequently a useful index of energy status in animals in different physiological states [43,44]. Russel and Wright [43] found a logarithmic regression relationship between plasma NEFA and energy intake in non-pregnant and non-lactating grazing cattle. In the current trial, NEFA were increased in *Desmanthus*-fed cattle (1.66-fold) corresponding to a 14% reduction in ME intake. Cortisol, being a product of the hypothalamic–pituitary–adrenal axis which coordinates physiological stress response, is a biomarker of stress in animals [46]. The lack of difference in cortisol between treatments showed that there was no stress due to the legume supplementation. 

### 3.4. Nitrogen Concentration in Animals Fed Lucerne and Desmanthus

Dietary protein in excess of animal requirement results in high concentrations of urea in the blood and urine. Urea-N is a fraction of total urinary N. It surges with an increase in dietary protein supply [47]. Ruminants retain, on average, between 10 to 45% of dietary N as milk or meat, with the majority excreted in feces and urine [48,49,50]. NH_3_ is produced in the rumen and hindgut by microorganisms and the catabolism of amino acids and other N-containing substrates in intermediary metabolism. Urea formation occurs mainly in the liver as a means of detoxification of NH_3_ present in systemic circulation. In cattle, net urea-N released by the liver accounts for 65% of increments in N intake [47]. On average, 47% of hepatic ureagenesis is returned to the gut through the portal-drained viscera [51].

The lack of difference in rumen NH_3_-N between the treatments was likely a consequence of similar CP in the treatments because dietary CP is correlated with the NH_3_-N concentration [29]. Rumen NH_3_-N herein was higher than in the previous study in 2018 (8 mg/dL) [16], reflecting the increased diet quality in the current trial with higher dietary CP and lower NDF. Plasma urea concentrations between 2.86 and 3.57 mmol/L were considered to be an optimal balance between energy intake and digestible protein. A plasma urea concentration exceeding 3.57 mmol/L was indicative of protein wastage [52]. The plasma urea concentration in our study was above 3.57 mmol/L, indicating protein wastage excreted in the feces and urine. The absence of any difference in plasma urea and rumen NH_3_-N concentrations reflected the negligible difference in dietary N. 

The higher fecal N in animals fed *Desmanthus* spp. than those on lucerne was reflective of the lower N in the diet and the potential effect of tannins. Previous studies had attributed higher fecal N to the presence of tannins as undigested tannin–protein complexes excreted in the feces [53] or to an enhancement in the absorption of essential amino acids from the small intestine, resulting in a shift of N excretion from urine to feces [54]. Grainger et al. [18] found a significant reduction in feed N lost to urine from 39, 26, and 22% by feeding dairy cows with an increasing amount of *Acacia mearnsii* CT in their diet at 0, 0.9, and 1.8% DMI, respectively. A more recent study by Lagrange et al. [19] showed that heifers grazing tanniferous legumes such as birdsfoot trefoil and sainfoin had lower urinary N concentrations (3.7 and 3.5 g/L) (6.0 g/L), but higher fecal N (34.5 and 35.5 g/kg) compared to the animals on lucerne (6.0 g/L and 30.5 g/kg for urinary and fecal N, respectively). They also showed that combining tanniferous legumes with lucerne improved urinary N which declined to 2.24 g/L. Fecal N is mainly in the organic form, which is less volatile than urinary N which is subject to nitrification and losses to ground water (leaching) [18]. Sordi et al. [21] stated that the emission factor for feces (0.15%) was lower than that of urine (0.26%). The urinary N concentration determined with the equation from Kohn et al. [25] were within the expected range for cattle (between 21 and 264 g/day). 

### 3.5. Effect of Polyethylene Glycol on Animal Performance, Rumen VFA, Plasma Metabolites, and Nitrogen Concentrations

The addition of PEG to the diet did not affect DMI, which corroborates the findings of Suybeng et al. [16], which reported no difference in DMI between PEG-supplemented and unsupplemented animals on diets containing 22% JCU1 or JCU4. However, it contradicts the study by Landau et al. [55] which showed that PEG supplementation may alleviate or even totally neutralize the negative effects of CT on DMI by feeding *Aspidosperma quebracho* to Holstein heifers. The decrease in DMI due to the presence of tannins has been attributed to its astringency property which makes the tissue either unpalatable to salivary proteins or by immobilizing enzymes [56]. Moreover, concentrations of tannins higher than 5% DM may be toxic to animals and induce desquamation and irritation of the intestinal mucosa, liver, and kidneys, resulting in lesions, ulcers, and even death [17]. Yisehak et al. [57] showed a significant increase in DMI by 9, 5, 10, and 6% with the addition of PEG in the diet of Zebu bulls fed 40% DM leaves of tannin-containing plants *Albizia gummifera*, *Grewia ferruginea*, *Prunus africana*, and *Syzygium guineense*, respectively. These plants contained 85, 55, 76, and 172 g CT/kg DM, respectively. However, due to the non-correlation between the efficacy of PEG addition and CT content, the authors suggested an evaluation of other factors that could help predict the efficacy of PEG such as the type of tannin or the interaction with other nutrients. Consequently, the lack of difference in DMI with the addition of PEG may be due to the tannin type, different interactions with other nutrients, or the tannin concentration in the diet. Furthermore, our results corroborate the findings of Suybeng et al. [16] which did not detect any differences in rumen VFA concentrations, except for an increase in iso-acids. The lower iso-acids concentration in the presence of tannins was attributed to the ability of tannins to bind proteins and protect them from ruminal deamination as iso-acids are derived from amino acid catabolism in the rumen [32,58,59]. 

A lower fecal N was expected in the present study in the animals supplemented with PEG, but the results showed a tendency for fecal N to be higher with PEG addition. It contradicts the findings by Mkhize et al. [60] that showed a decrease in fecal N with the addition of PEG in the diet of grazing goats compared to when they were supplemented with water or CT. The higher dietary N during the PEG period might explain the higher fecal N concentration in the presence of PEG as N excretion by beef cattle is positively correlated with N intake in the diet [61]. 

## 4. Materials and Methods

This study was conducted at the Commonwealth Scientific and Industrial Research Organisation (CSIRO) Lansdown Research Station, Queensland, Australia (19°59′ S, 146°84′ E). All procedures complied with the Australian Code for the Care and Use of Animals for Scientific Purposes (Eighth edition, 2013) as approved by the CSIRO Queensland Animal Ethics Committee (Permit Number 2019-32).

### 4.1. Animals and Treatment

**Figure 1:**
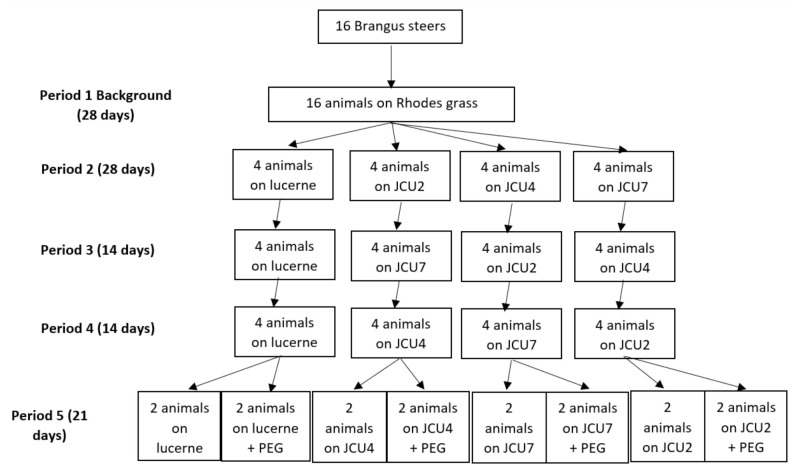
The experimental design.

Sixteen yearling Brangus steers were fed a basal diet of Rhodes hay supplemented with forage legumes comprising lucerne or one of the following three *Desmanthus* cultivars: *D. virgatus* cv. JCU2, *D. bicornutus* cv. JCU4, and *D. leptophyllus* cv. JCU7. A completely randomized block experimental design was utilized. Steers had an average LW of 232 ± 6 kg and were randomly allocated into four blocks by weight. One block was allocated to lucerne throughout the study, while the remaining three blocks were allocated to a different *Desmanthus* cultivar in each of the three periods as depicted in Figure 1. This design was chosen to avoid the expected nutritional perturbations associated with changing from lucerne to *Desmanthus*. The first 28 days of the trial constituted a background period where all the animals were fed Rhodes grass (*Chloris gayana*) hay only. The background period was followed by a period of 28 days including 10 days to allow the animals to adapt to the Rhodes grass plus lucerne hay or one of the *Desmanthus* cultivars at a planned level of inclusion of 30% DM. Samples of each *Desmanthus* cultivar and lucerne were sent to Feed Central (Charlton, QLD, Australia) for NIRS analysis to match the targeted CP of 21%. Thus, the overall lucerne content of the diet was 21.3% on a DM basis. The following periods lasted 14 days to allow the animals on *Desmanthus* to adapt to the new *Desmanthus* species. In every period, each steer on *Desmanthus* received a different *Desmanthus* cultivar. At the conclusion of the study, animals remained on their respective diets for a further 21 days with half of the animals on each treatment supplemented with polyethylene glycol (PEG 4000, Redox Pty Ltd, Minto, NSW, Australia) at 160 g/kg DM of *Desmanthus* or lucerne to nullify the bioactivity of tannins. This period included a 5-day adaptation period where the animals were fed an increasing level of PEG (50 g/day) before reaching their full consumption amounts. The adaptation period to the diet was considered adequate as it was within the 10 to 14 days range suggested by Cochran and Galyean [62]. All animals were fed ad libitum to 10% refusals over the first seven days of each period. Thereafter, intake was reduced to 90% of ad libitum. The three *Desmanthus* cultivars were harvested fresh using a crop chopper (New Holland Model 38 Crop-Chopper^®^, Haryana, India) on alternate days from a farm located 20 min away from the research station (19°63′ S, 146°90′ E). The fresh *Desmanthus* was consistently harvested at 7:00 am between four- and six-week regrowth to capture the vegetative stage of maturity to minimize differences in nutritive value between the cultivars. The *Desmanthus* was stored at 5 °C in a cool room prior to being fed out. The *Desmanthus* was mixed with chopped Rhodes grass hay (Roto grind model 760, Burrows Enterprises, LLC, Greeley, CO, USA) before being fed to the steers. The lucerne hay was also chopped (Roto grind model 760, Burrows Enterprises, LLC, Greeley, CO, USA) and mixed with Rhodes grass at the same time as the *Desmanthus* treatments immediately before feeding. Diets were fed out once daily between 9:30–10:00 am and all experimental steers had continuous access to reticulated water and mineral blocks (Trace element Northern, Olsson’s, Yennora, NSW, Australia).

### 4.2. Feed Chemical Composition and Analysis

Samples of offered (individual forages) and uneaten feeds were taken daily in three consecutive days of the end of each period. Samples were sent for near-infrared reflectance spectroscopy (NIRS) analysis using a scanning monochromator (model 6500, NIRSystem, Inc., Silver Spring, MD, USA) and calibration equations developed by CSIRO Agriculture [63] using ISI Software (Infrasoft International, Port Matilda, PA, USA) as described by Durmic et al. [64]. Samples of Rhodes grass, lucerne, JCU2, JCU4, and JCU7 were also sent to FeedTest (Agrifood Technology, Victoria, Australia) for wet chemistry analyses in order to compare the NIRS and wet chemistry results. The DM (method 934.01) and CP (method 954.01) contents of the samples were determined according to the procedures of the Association of Official Analytical Chemist (2000) [65]. The heat-stable α-amylase-treated neutral detergent fiber (NDF) and (acid detergent fiber) ADF contents were analyzed according to the procedure described by Van Soest [66]. In vitro DM digestibility (DMD) was determined using a modified pepsin-cellulase technique described by Clarke et al. [67].

Metabolizable energy (ME) was determined as DMD × 0.172−1.707 [24] from the NIRS data.

### 4.3. Dry Matter Intake and Liveweight Gain

The LW of each animal was recorded automatically (Gallagher Smart TSI, Melbourne, Victoria, Australia) weekly prior to feeding to determine the daily LW gain. Individual DMI was determined by the difference between offered and residual feed after 24 h. Individual daily intakes were recorded throughout the study to determine treatment group DMI. These values were used to calculate the DMI expressed as % of LW. Feed conversion ratio was calculated as the average of DMI for the 3 periods (periods 2, 3, and 4) on legumes without PEG supplementation divided by the daily LW gain during the same periods.

### 4.4. F.NIRS Estimates of Diet Quality and Estimation of Urinary N

Fecal samples were collected from the rectum of each steer 3 h post-feeding at the end of each period. The samples were dried in a fan-forced oven at 60 °C and ground through a Tecator Cyclotec 1093 (FOSS, Hillerød, North Zealand, Denmark) fitted with a 1-mm screen. A monochromator fitted with a spinning cup module (NIRSystems FOSS 6500, Hilleroed, Denmark) was used to scan fecal NIR spectra at the CSIRO Floreat laboratory (Floreat, WA, Australia). All spectra analyses, data manipulation, and spectra calibrations were done with ISI software. The calibration equation for predicting the dietary CP concentration and dry matter digestibility (DMD) (R^2^ = 0.92) published by Coates and Dixon [63], Coates [68], and Coates and Dixon [69] were used to estimate diet quality. 

Urinary N concentration was estimated using the equation from Kohn et al. [25] as follows: Urinary N (g/day) = CR × BUN × LW with CR representing the clearance rate (liters of blood cleared completely of urea per day which is estimated to be equal to 1.3 for cattle), BUN = blood urea nitrogen (g/L) which is equal to plasma urea divided by 357.1 and LW = liveweight (kg).

### 4.5. Rumen Collection and Volatile Fatty Acids Analysis

Rumen fluid was collected three hours after feeding on the last day of each period. Rumen fluid samples were collected through an oral stomach tube using a reinforced plastic suction tube (approximately 3 cm in diameter). A hand pump was used to extract 100–200 mL of rumen fluid from the ventral sac. Rumen fluid pH was immediately measured using a pH meter and a sub-sample taken, mixed with fresh 20% metaphosphoric acid (4:1) and frozen at −80 °C for VFA and NH_3_-N analyses. Rumen fluid concentrations of short chain fatty acids (acetate, propionate, n-butyrate, iso-butyrate, iso-valerate, n-valerate, and n-caproate) were measured by gas chromatography as described by Gagen et al. [70]. NH_3_-N concentration was determined by the colorimetric method of Chaney and Marbach [71].

### 4.6. Blood Sample Collection and Plasma Metabolite Analysis

Blood samples (10 mL) were collected from each experimental steer 3 h after morning feeding at the end of each period. All samples were collected using jugular venipuncture. These were stored in BD Vacutainer^®^ Lithium Heparin Tubes (Becton, Dickinson and Company, Belliver Way, Belliver Industrial Estate, Plymouth, Devon, UK), immediately chilled in an ice-containing esky and later centrifuged at 2500 rpm for 20 min at 4 °C (Allegra^®^ 6 Series and Spinchron™ R Centrifuges, Beckman Coulter, Inc., Brea, CA, USA). The plasma was separated from the serum and sub-samples of the plasma were taken and stored at −80 °C until analysis. All samples were analyzed for plasma metabolite concentrations at the Veterinary Clinical Pathology Laboratory of the College of Public Health, Medical and Veterinary Sciences at James Cook University, Townsville, Queensland, Australia. Plasma glucose was analyzed by an enzymatic UV test (hexokinase method), plasma urea was analyzed by a kinetic UV test, and non-esterified fatty acids (NEFA) were measured with the FA115 kit of Randox (Randox Laboratories Ltd., Crumlin, County Antrim, UK). The three analyses were done on Beckman Coulter AU480 Analyzer (Beckman Coulter, Inc., Brea, CA, USA). Cortisol was analyzed using Immulite 1000 Systems analyzer (Siemens, Erlangen, Germany) and a solid-phase, competitive chemiluminescent enzyme immunoassay.

### 4.7. Statistical Analyses

All data were analyzed using R (Rstudio version 1.3.1056, R Core Team (2013). R: A language and environment for statistical computing. R Foundation for Statistical Computing, Vienna, Austria, ISBN 3-900051-07-0, URL http://www.R-project.org/) with the ‘dplyr’ [72], ‘nlme’ [73], ‘lme4’ [74], ‘car’ [75], and ‘multcomp’ [76] packages. Effects were considered significant at *p* < 0.05 and considered as “tendency” at *p* < 0.06.

A linear mixed model procedure was utilized to compare the chemical compositions and their effects on intake, daily LW gain, rumen VFA and plasma metabolite profiles between the four different treatments (lucerne, JCU2, JCU4 and JCU7 diet). DMI, LW, daily LW gain, DMI/kg LW, feed conversion ratio, rumen VFA and plasma metabolites, dietary N, N intake, rumen NH_3_-N, plasma urea, fecal and urinary N were the dependent variables, while the four treatments were the fixed effects and individual animals nested within groups were the random effects. The same model was used to analyze the effect of PEG on intake, rumen VFA, plasma metabolite profiles and N concentrations except that only the data from the animals on the *Desmanthus* diet in period 5 were analyzed and the fixed effect was the presence or absence of PEG, hence the use of 12 animals in period 5 statistical analysis. The models were based on the restricted maximum likelihood (REML) technique. Pearson’s product–moment correlation analysis was also conducted to estimate correlations and *p*-values between NIRS and wet chemistry determined nutritive values for the forage samples. Crude protein, ADF, NDF, DMD, and ME were the dependent variables and the analytic method the fixed effect.

## 5. Conclusions

The utilization of N in *Desmanthus* diets differed from that in lucerne. *Desmanthus virgatus* (JCU2), *Desmanthus bicornutus* (JCU4), and *Desmanthus leptophyllus* (JCU7) showed broadly similar results regarding animal performance, plasma metabolite and VFA profiles and N concentrations. The presence of tannins reduced proteolysis in the rumen, as evidenced by lower rumen NH_3_-N, and contributed to higher N flow to the lower tract, as evidenced by higher fecal N concentration compared to lucerne. The inclusion of PEG to nullify the tannin effects induced an increase in rumen iso-acids. *Desmanthus* spp. were of a lower quality with higher fiber and lower energy content than lucerne which resulted in lower dry matter intake and animal performance. Nonetheless, the inclusion of *Desmanthus* in diets has the potential to increase the performance of tropical beef cattle in Northern Australia, possibly due to a better N utilization attributable to the presence of tannins. These findings could potentially contribute to increased animal production and performance in the drier parts of Northern Australia. Further in vivo investigation is needed to better understand the impact of tannins in *Desmanthus* on N utilization and evaluation of outdoor methane emissions in Northern Australian beef cattle supplemented with *Desmanthus*.

## Figures and Tables

**Table 1:** Chemical compositions (mean ± s.e.) of dietary components predicted by near infrared reflectance spectroscopy.

Variable	Rhodes Grass	Lucerne	JCU2 (*D. virgatus*)	JCU4 (*D. bicornutus*)	JCU7 (*D. leptophyllus*)	*Desmanthus* spp. ^2^
DM (%)	84.0 ± 0.94	84.0 ± 1.16	32.3 ± 1.61	30.7 ± 1.26	34.2 ± 0.93	32.3 ± 0.78
CP (% DM)	8.8 ± 0.19	15.1 ± 0.61	10.3 ± 0.99	13.0 ± 0.85	10.6 ± 0.80	11.3 ± 3.65
ADF (% DM)	42.8 ± 0.43	37.5 ± 0.73	44.5 ± 1.33	40.4 ± 1.10	43.4 ± 1.00	42.8 ± 0.70
NDF (% DM)	73.8 ± 0.41	49.6 ± 0.79	57.5 ± 1.38	53.1 ± 1.34	58.5 ± 1.20	56.4 ± 0.81
DMD (%)	50.6 ± 0.56	65.2 ± 1.08	47.8 ± 2.57	51.7 ± 1.55	49.4 ± 1.41	49.6 ± 0.011
ME (MJ/kg DM) ^1^	7.0 ± 0.10	9.5 ± 0.19	6.5 ± 0.44	7.2 ± 0.27	6.8 ± 0.24	7.4 ± 0.19

^1^ Estimated as DMD × 0.172 − 1.707 [24], ^2^ average of the three *Desmanthus* spp. DM = dry matter, CP = crude protein, ADF = acid detergent fiber, NDF = neutral detergent fiber, DMD = dry matter digestibility, ME = metabolizable energy.

**Table 2:** Comparative chemical composition of the experimental dietary component determined by wet chemistry and NIRS.

Variable	Rhodes Grass		Lucerne		JCU2 (*D. virgatus*)		JCU4 (*D. bicornutus*)		JCU7 (*D. leptophyllus*)		*Desmanthus* spp. ^2^		*p*-Value
	Wet Chemistry	NIRS	Wet Chemistry	NIRS	Wet Chemistry	NIRS	Wet Chemistry	NIRS	Wet Chemistry	NIRS		r between NIR and Wet Chemistry Values	
CP (% DM)	9.8	9.0	17.0	16.6	19.6	13.8	17.4	16.7	14.3	7.5	17.1	0.71	0.18
ADF (% DM)	40.0	44.0	35.3	36.3	21.9	39.7	30.9	35.9	30.5	46.7	27.8	0.14	0.82
NDF (% DM)	72.7	74.7	47.4	48.5	33.0	47.3	43.5	49.8	45.8	61.8	40.8	0.89	0.04
DMD (%)	45.3	48.4	58.8	70.0	50.1	60.6	41.8	59.0	43.2	48.3	45.1	0.79	0.11
ME (MJ/kg DM) ^1^	6.1	6.6	8.4	10.3	6.9	8.7	5.5	8.4	5.8	6.6	7.9	0.79	0.11

^1^ Estimated as DMD × 0.172 − 1.707 [24], ^2^ average of the three *Desmanthus* spp. calculated using the wet chemistry results. DM = dry matter, CP = crude protein, ADF = acid detergent fiber, NDF = neutral detergent fiber, DMD = dry matter digestibility, ME = metabolizable energy.

**Table 3:** Nutritive value (mean ± s.e.) of diets containing lucerne and *Desmanthus* spp. cultivars determined by NIRS.

Variable	Lucerne	JCU2	JCU4	JCU7	*Desmanthus* spp. ^2^	SEM	*p*-Value
CP (% DM)	10.2	9.2	10.1	9.4	9.6	0.17	0.08
ADF (% DM)	41.3 a	41.9 b	41.9 b	42.8 b	42.6	0.62	0.01
NDF (% DM)	68.4	68.7	67.2	68.8	68.3	0.74	0.13
ME (MJ/kg DM) ^1^	7.6 a	7.1 b	7.1 b	7.0 b	7.0	0.06	0.01

^1^ Estimated as DMD × 0.172 − 1.707 [24], ^2^ average of the three *Desmanthus* spp. DM = dry matter, CP = crude protein, ADF = acid detergent fiber, NDF = neutral detergent fiber, ME = metabolizable energy, SEM = standard error of the mean. Means within the same row without the same alphabetical characters (a, b) represent statistical differences (*p* < 0.05).

**Table 4:** Initial and final DMI, LW, daily gains, and feed conversion ratio of steers fed lucerne and *Desmanthus* spp.

Variable	Lucerne	*Desmanthus* spp.	SEM	*p*-Value
Initial DMI (kg/day)	5.5	5.4	0.12	0.67
Final DMI (kg/day)	6.5	6.1	0.14	0.03
Initial LW (kg)	270.7	275.0	0.04	0.95
Final LW (kg)	320.0	303.4	0.03	0.04
Daily LW gain (kg/day)	0.6	0.34	0.04	0.01
DMI/kg LW (%)	2.0	2.0	0.03	0.98
Feed conversion ratio	10.6	22.9	4.70	0.19

DMI = dry matter intake, LW = liveweight, SEM = standard error of the mean.

**Table 5:** Rumen VFA and plasma metabolites of steers fed lucerne and *Desmanthus* spp.

Variable	Lucerne	JCU2	JCU4	JCU7	*Desmanthus* spp.	SEM	*p*-Value
**Rumen volatile fatty acids**							
Total VFA (mg/100 dL)	65.2 a	60.2 ab	57.0 ab	51.5 b	56.4	1.64	0.01
Acetate (molar %)	75.4	76.5	75.9	76.8	76.4	0.27	0.17
Propionate (molar %)	14.5	13.9	14.5	14.0	14.2	0.15	0.34
Acetate/propionate ratio	5.2	5.5	5.3	5.5	5.4	0.08	0.34
Iso-Butyrate (molar %)	0.97 a	0.81 ab	0.76 b	0.80 b	0.79	0.02	0.01
n-Butyrate (molar %)	7.0	6.8	6.9	6.6	6.8	0.13	0.63
Iso-Valerate (molar %)	1.0 a	0.87 ab	0.83 b	0.86 ab	0.85	0.02	0.02
n-Valerate (molar %)	0.95	0.89	0.93	0.81	0.88	0.04	0.65
n-Caproate (molar %)	0.15	0.16	0.17	0.17	0.17	0.01	0.87
pH	7.0	7.0	7.1	7.2	7.1	0.03	0.10
**Plasma metabolites**							
Glucose (mmol/L)	4.2	4.1	4.2	4.1	4.1	0.05	0.08
NEFA (mmol/L)	0.053 a	0.074 ab	0.11 b	0.081 ab	0.088	0.01	0.01
Cortisol (nmol/L)	28.0	30.3	23.7	27.6	24.3	3.39	0.97

VFA = volatile fatty acids, SEM = standard error of the mean, NEFA = non-esterified fatty acids. Means within the same row without the same alphabetical characters (a,b) represent statistical differences (*p* < 0.05).

**Table 6:** Dietary, plasma, rumen, fecal, and urinary N metabolism in steers supplemented with lucerne and *Desmanthus* spp.

Variable	Lucerne	JCU2	JCU4	JCU7	*Desmanthus* spp. ^2^	SEM	*p*-Value
Diet N (% DM)	1.6	1.5	1.6	1.5	1.5	0.03	0.30
Diet N by F.NIRS (% DM)	2.4 a	2.2 b	2.2 b	2.2 b	2.2	0.04	0.01
N intake (g/day)	111.8 a	92.8 b	101.7 ab	92.0 b	95.8	2.77	0.01
Rumen NH3-N (mg/dL)	17.6	15.5	16.4	15.6	15.8	0.46	0.32
Plasma urea (mmol/L)	5.3	5.4	5.6	5.5	5.5	0.16	0.96
Fecal N (% DM)	1.8 a	1.9 b	2.0 b	2.1 c	2.0	0.03	0.01
Urinary N (g/day) ^1^	59.3	58.5	59.3	60.0	59.3	1.92	0.64

^1^ Estimated as CR x BUN x LW (with CR = clearance rates of 1.3 for cattle, BUN = blood urea nitrogen, LW = liveweight) [25]. ^2^ Average of the three *Desmanthus* spp. N = nitrogen, SEM = standard error of the mean. Means within the same row without the same alphabetical characters (a, b, c) represent statistical differences (*p* < 0.05).

**Table 7:** Effect of polyethylene glycol on animal performance, rumen VFA, plasma metabolites, and nitrogen concentrations.

Variable	*Desmanthus* spp.		SEM	*p*-Value
	No PEG	PEG		
**Animal performance**				
DMI (kg/day)	5.6	6.2	0.21	0.20
**Rumen volatile fatty acids**				
Total VFA (mg/100 dL)	37.7	40.9	3.60	0.69
Acetate (molar %)	80.1	77.9	0.50	0.06
Propionate (molar %)	12.2	13.0	0.28	0.23
Acetate/propionate ratio	6.6	6.0	0.18	0.17
Iso-Butyrate (molar %)	0.63	0.92	0.05	0.01
n-Butyrate (molar %)	5.4	6.0	0.16	0.18
Iso-Valerate (molar %)	0.7	1.0	0.06	0.01
n-Valerate (molar %)	0.84	0.98	0.04	0.04
n-Caproate (molar %)	0.14	0.16	0.01	0.54
pH	7.0	7.3	0.08	0.11
**Plasma metabolites**				
Glucose (mmol/L)	4.2	4.4	0.11	0.43
NEFA (mmol/L)	0.12	0.12	0.02	0.99
Cortisol (nmol/L)	25.9	22.2	5.12	0.73
**Nitrogen concentrations**				
Diet N (% DM)	1.5	1.6	0.05	0.36
Diet N by F.NIRS (% DM)	2.1	2.2	0.05	0.98
N intake (g/day)	99.3	108.0	6.48	0.51
Rumen NH3-N (mg/dL)	15.6	15.5	1.27	0.95
Plasma urea (mmol/L)	5.6	6.1	0.28	0.43
Fecal N (% DM)	2.1	2.2	0.05	0.05
Urinary N (g/day) ^1^	62.6	73.4	4.36	0.35

^1^ Estimated as CR × BUN × LW (with CR = clearance rates of 1.3 for cattle, BUN = blood urea nitrogen) [25]. PEG = polyethylene glycol, SEM = standard error of the mean, DMI = dry matter intake, N = nitrogen, VFA = volatile fatty acids, NEFA = non-esterified fatty acids.

## Data Availability

Data available on request.
